# An Account of the Salutary Effect of Mercury in a Case Attended with Symptoms of Hydrocephalus Internus

**Published:** 1785

**Authors:** 


### THE LONDON MEDICAL JOURNAL,

FOR THE YEAR 1785,

### PART THE SECOND.

### SECTION I.

### ESSAYS AND OBSERVATIONS.

I. *An Account of the ſalutary Effect of Mercury in a Caſe attended with Symptoms of Hydrocephalus Internus. Communicated in a Letter to Dr.* Simmons, *by* James Moſeley, M. D. *Phyſician at Ludlow.*

NOTWITHSTANDING mercurial medicines have been of late years recommended in the caſe of Hydrocephalus Internus, yet as the practice is not general, and as many may be intimidated from uſing them in ſufficiently large quantity, until the ſafety of the practice is confirmed by experience and repeated practice, the incloſed caſe, ſucceſsfully treated, is a ſtrong proof of both the ſafety and utility of the practice. If you think the manner in which I have drawn it up compatible with the judicious plan of the Medical Journal, I beg leave, by your means, to ſee it inſerted in that work.

### CASE.

UPON the 23d of September, 1784, I was called to viſit the ſon of a reputable tradeſman in this town, between two and three years of age, who, I was informed, had been ill fourteen or fifteen days of a briſk fever, with conſiderable fluſhings in his face, and violent pain in the head; that the two laſt days he had voided his ſtools and urine inſenſibly, and was now totally deprived of his ſight.

I found my little patient's pulſe rather ſlow and weak, with ſome irregularity in its ſtrokes; his countenance was very florid; the pupils of his eyes were exceedingly dilated, and upon a candle being preſented very near, they did not ſhew the leaſt irritability or tendency to contract; and upon examination, the ſagittal ſuture appeared ſomewhat ſeparated.

Under ſuch a train of unfavourable ſymptoms, I entertained very little hopes of being of ſervice; being convinced, however, of the

ſhort

ſhort time there was left to try the powers of medicine, and being anxious for my little patient, I was determined to put him upon a mercurial courſe, and accordingly directed half a drachm of the unguentum mercuriale fortius to be rubbed in upon the thighs, and repeated in four hours, alſo one grain of calomel to be given every four hours; and a bliſter to be applied to the head.

The next morning, (Sept. 24th) the ſymptoms appeared as yeſterday. He had had no ſtool, but had voided his urine inſenſibly; one drachm of the ointment had been rubbed in, and four grains of calomel given. He ſhewed no ſenſibility of the bliſter, which had riſen but little. I directed a drachm more of the ointment to be rubbed in, and a grain and a half of calomel to be given every four hours.

The night was very reſtleſs, but the following day, (Sept. 25th) there was a conſiderable alteration for the better; the pupils poſſeſſed a conſiderable contractile power, and he was ſenſible of voiding his urine. The mercurial medicines were ordered to be omitted.

Sept. 26. He continued better.

The night was very much diſturbed, and the next day (Sept. 27th) he was evidently worſe; the pupils were more dilated, and lefs irritable. From this unfavourable appearance, fufpecting I had laid afide the mercury too foon, I refolved to give it a farther trial, and therefore ordered half a drachm more of the ointment to be rubbed in immediately, and one grain and a half of calomel to be given, and both to be repeated in the evening.

Sept. 28. The fymptoms being fomewhat aggravated, a drachm more of the ointment was ordered to be rubbed in in the courfe of the day, and one grain of calomel to be given every four hours.

Sept. 29, we had for a fecond time a more favourable appearance. Half a drachm of the ointment was ordered to be rubbed in, and one grain and a half of calomel to be given, and repeated in the evening.

Sept. 30, he continued gaining ground; and upon the firft of October the mercurials were omitted, and the bark was given by way of a tonic, and continued for fome days, when all medicines were laid afide.

Through the whole of this cafe, there was a coftivenefs, which I was averfe to remove, left the mercury fhould run off by ftool.

With

With reſpect to the nature of the diſeaſe, I think there can remain no doubt; and it ſeems equally evident that the cure depended on the Mercury. The aggravation of the ſymptoms on the 27th and 28th, there is no doubt, proceeded from laying aſide the mercury too ſoon. In this opinion, I am confirmed by their becoming more favourable, and regularly diſappearing as ſoon as it could, from the ſecond application, be ſuppoſed to act upon the ſyſtem. The number of deſperate ſymptoms that preſented, encouraged me in ſo liberal a uſe of mercurials; and the ſucceſs that followed, ſhows the ſafety and propriety of the practice.

It may not be foreign to obſerve, that the child never voided one worm during his illneſs or ſince; and that there did not appear any fœtor of the breath, redneſs of the gums, ptyaliſm or ſymptom of a ſalival tendency.

I ſhall conclude with obſerving, that the child continues perfectly well, excepting that the pupil of the right eye ſeems ſomewhat more dilated than that of the left, the ſagittal ſuture being perfectly cloſed.

*Ludlow,*
February 24, 1785.

